# A meta-analysis of genome-wide data from five European isolates reveals an association of *COL22A1*, *SYT1*, and *GABRR2 *with serum creatinine level

**DOI:** 10.1186/1471-2350-11-41

**Published:** 2010-03-11

**Authors:** Cristian Pattaro, Alessandro De Grandi, Veronique Vitart, Caroline Hayward, Andre Franke, Yurii S Aulchenko, Asa Johansson, Sarah H Wild, Scott A Melville, Aaron Isaacs, Ozren Polasek, David Ellinghaus, Ivana Kolcic, Ute Nöthlings, Lina Zgaga, Tatijana Zemunik, Carsten Gnewuch, Stefan Schreiber, Susan Campbell, Nick Hastie, Mladen Boban, Thomas Meitinger, Ben A Oostra, Peter Riegler, Cosetta Minelli, Alan F Wright, Harry Campbell, Cornelia M van Duijn, Ulf Gyllensten, James F Wilson, Michael Krawczak, Igor Rudan, Peter P Pramstaller, the EUROSPAN consortium

**Affiliations:** 1 Institute of Genetic Medicine, European Academy Bozen/Bolzano (EURAC), Bolzano, Italy - Affiliated Institute of the University Lübeck, Lübeck, Germany; 2 MRC Human Genetics Unit, Institute of Genetics and Molecular Medicine, Edinburgh, UK; 3 Institute for Clinical Molecular Biology, Christian-Albrechts-University Kiel, Kiel, Germany; 4 Genetic Epidemiology Unit, Departments of Epidemiology and Clinical Genetics, Erasmus MC, 3000 CA Rotterdam, the Netherlands; 5 Department of Genetics and Pathology, Rudbeck laboratory, Uppsala University, SE-751 85, Uppsala, Sweden; 6 Centre for Population Health Sciences, University of Edinburgh Medical School, Teviot Place, Edinburgh EH8 9AG, UK; 7 Andrija Stampar School of Public Health, University of Zagreb Medical School, Rockefellerova 4, 10000 Zagreb, Croatia; 8 Gen-info Ltd, Ruzmarinka 17, 10000 Zagreb, Croatia; 9 Popgen biobank, Christian-Albrechts-University Kiel, Kiel, Germany; 10 Institute for Experimental Medicine, Christian-Albrechts University Kiel, 24105 Kiel, Germany; 11 Croatian Centre for Global Health, University of Split Medical School, Soltanska 2, 21000 Split, Croatia; 12 Institute for Clinical Chemistry and Laboratory Medicine, Regensburg University Medical Center, D-93053 Regensburg, Germany; 13 Institute of Human Genetics, Technical University of Munich, Munich, Germany; 14 Institute of Human Genetics, Helmholtz Zentrum München, German Research Center for Environmental Health (GmbH), Ingolstaedter Landstr 1, D-85764 Neuherberg, Germany; 15 Hemodialysis Unit, Hospital of Merano, Merano, Italy; 16 Institute of Medical Informatics and Statistics, Christian-Albrechts-University, Kiel, Germany; 17 Department of Neurology, University of Lübeck, Lübeck, Germany; 18 Department of Neurology, Central Hospital, Bolzano, Italy

## Abstract

**Background:**

Serum creatinine (S_CR_) is the most important biomarker for a quick and non-invasive assessment of kidney function in population-based surveys. A substantial proportion of the inter-individual variability in S_CR _level is explicable by genetic factors.

**Methods:**

We performed a meta-analysis of genome-wide association studies of S_CR _undertaken in five population isolates ('discovery cohorts'), all of which are part of the European Special Population Network (EUROSPAN) project. Genes showing the strongest evidence for an association with S_CR _(candidate loci) were replicated in two additional population-based samples ('replication cohorts').

**Results:**

After the discovery meta-analysis, 29 loci were selected for replication. Association between S_CR _level and polymorphisms in the collagen type XXII alpha 1 (*COL22A1*) gene, on chromosome 8, and in the synaptotagmin-1 (*SYT1*) gene, on chromosome 12, were successfully replicated in the replication cohorts (p value = 1.0 × 10^-6 ^and 1.7 × 10^-4^, respectively). Evidence of association was also found for polymorphisms in a locus including the gamma-aminobutyric acid receptor rho-2 (*GABRR2*) gene and the ubiquitin-conjugating enzyme E2-J1 (*UBE2J1*) gene (replication p value = 3.6 × 10^-3^). Previously reported findings, associating glomerular filtration rate with SNPs in the uromodulin (*UMOD*) gene and in the schroom family member 3 (*SCHROOM3*) gene were also replicated.

**Conclusions:**

While confirming earlier results, our study provides new insights in the understanding of the genetic basis of serum creatinine regulatory processes. In particular, the association with the genes *SYT1 *and *GABRR2 *corroborate previous findings that highlighted a possible role of the neurotransmitters GABA_A _receptors in the regulation of the glomerular basement membrane and a possible interaction between GABA_A_receptors and synaptotagmin-I at the podocyte level.

## Background

In epidemiological population-based surveys, serum creatinine (S_CR_) represents the most important biomarker for a quick and non-invasive assessment of kidney function, allowing estimation of the glomerular filtration rate (GFR) [1]. At the same time, an increased S_CR _level has also been recognized as a risk factor for adverse outcomes in patients hospitalized for cardiac surgery or heart failure [2]. A substantial proportion of the inter-individual variability in S_CR _level is explicable by genetic factors. Twin [3] and pedigree-based studies [4-7] yielded heritability estimates ranging from 0.19 to 0.53, with higher values observed in subjects not treated for hypertension [4]. A large number of genetic loci have emerged from genome-wide linkage analyses as being related to variation in S_CR _level or in S_CR_-based estimates of both GFR and creatinine clearance [4-15]. Focusing upon quantitative renal phenotypes, a recent GWA study [16] and a subsequent replication study [17] identified variants in the 5,10-methenyltetrahydrofolate synthetase (*MTHFS*) gene to be associated with chronic kidney disease (CKD; defined as GFR < 60 ml/min/1.73 m^2^). More recently, variants in the uromodulin (*UMOD*) gene have been shown to be associated with GFR and CKD independent of age, sex, hypertension, and diabetic status [18]. In the same study, SNPs in the schroom family member 3 (*SCHROOM3*) gene, the glycine amidinotransferase (*GATM*)-spermatogenesis associated 5-like 1 (*SPATA5L1*) locus, and in the jagged 1 (*JAG1*) gene were also found to be associated with GFR [18].

We have reported linkage between S_CR _level and a region at 22q13 containing the myosin heavy chain 9 non-muscle (*MYH9*) gene [4], a locus that had been associated with non-diabetic end-stage renal disease (ESRD) [19,20] and glomerulosclerosis [21] before. This linkage was detected in families from three isolated European populations participating in the European Special Population Research Network (EUROSPAN). Here, we have performed a genome-wide association analysis of S_CR _level combining data from all five EUROSPAN populations, with S_CR _re-measured using an enzymatic method in one and the same central laboratory. We have performed population-specific GWA studies and subjected the results to an inverse-variance, fixed effects meta-analysis. Selected candidate regions were then tested in two additional, population-based samples from Europe.

## Methods

### Study samples

For all EUROSPAN and replication studies, written informed consent was obtained from all participants and all protocols were approved by the institutional ethical review committees of the participating centres.

### Eurospan

The EUROSPAN project http://homepages.ed.ac.uk/s0565445/index.html was initiated in 2006 and involves five population isolates from Italy, Croatia, Scotland, Sweden, and the Netherlands. The project aims at assessing the genetic structure of European isolates and at identifying genes underlying common traits, taking advantage of the genetic and environmental homogeneity that usually characterizes population isolates. In the current context, according to Neel [22], with population isolates we mean "*secondary isolates*", i.e. groups that, for some reasons, detached or were detached from larger populations. In particular, EUROSPAN cohorts were derived from small population samples which have grown slowly, with little recruitment from outside the groups.

The *ERF *study is a family-based project including over 3000 participants that originated from 22 couples living in the Rucphen region of the Netherlands in the 19^th ^century [23-25]. All descendants of these people were invited to visit a clinical research center in the region, where they were examined in person and where blood was taken after fasting. Height and weight were measured for each participant. All participants filled out a questionnaire on risk factors.

The *MICROS *study is part of the genomic health care program 'GenNova' and was carried out in three villages of the Val Venosta, South Tyrol (Italy), in 2001-2003. It comprised members of the populations of Stelvio, Vallelunga and Martello. A detailed description of the *MICROS *study can be found elsewhere [26]. Briefly, study participants were volunteers from three isolated villages located in the Italian Alps, in a German-speaking region bordering upon Austria and Switzerland. Owing to geographical, historical and political reasons, the entire region experienced a prolonged period of isolation from surrounding populations. Information on the participants' health status was collected through a standardized questionnaire. Laboratory data were obtained from standard blood analyses. Genotyping was performed on >1400 participants, with 1334 of them suitable for analysis after data cleaning.

The Northern Swedish Population Health Study (*NSPHS*) is a family-based study including a comprehensive health assessment and the collection of data on family structure, lifestyle, diet, medical history and of samples for laboratory analyses [27,28]. Participants came from the northern part of the Swedish mountain region (County of Norrbotten, Parish of Karesuando). Historic population accounts show that little migration or population changes have occurred in this area over the last 200 years.

The Orkney Complex Disease Study (*ORCADES*) is an ongoing, family-based and cross-sectional study in the isolated Scottish archipelago of Orkney [29]. Genetic diversity in this population is reduced in comparison to Mainland Scotland, consistent with high levels of historical endogamy. Participants were aged 18-100 years and came from a subgroup of ten islands. Fasting blood samples were collected and over 200 health-related phenotypes and environmental exposures were measured in each individual.

The *Vis *study includes 986 unselected Croatians, aged 18-93 years, who were recruited during 2003 and 2004 from the villages of Vis and Komiza on the Dalmatian island of Vis [30,31]. The settlements on Vis island have a unique history and remained isolated from other villages and the outside world for centuries. Participants were phenotyped for 450 disease-related quantitative traits. Biochemical and physiological measurements were performed, detailed genealogies reconstructed, questionnaires on lifestyle and environmental exposures collected, and blood samples and lymphocytes extracted and stored for further analyses.

All DNA samples were genotyped on Illumina Infinium HumanHap300 v2 SNP bead microarrays according to the manufacturer's instructions, except for samples from Vis for which version 1 was used (the Vis samples had 311,398 SNPs genotyped in common with the other populations).

For all five studies, serum or plasma creatinine was measured at the Institute for Clinical Chemistry and Laboratory Medicine, Regensburg University Medical Center, Germany, using an enzymatic photometric assay on an ADVIA1650 clinical chemistry analyzer (Siemens Healthcare Diagnostics GmbH, Eschborn, Germany) [32]. The number of individuals with available creatinine, sex, and age information is reported for each study in Table 1.

**Table 1:** Characteristics of studies and study participants.

	EUROSPAN					REPLICATION STUDIES	
**Study name**	**ERF**	**MICROS**	**NSPHS**	**ORCADES**	**VIS**	**popgen**	**Korcula**

**Nationality**	The Netherlands	Italy	Sweden	UK	Croatia	Germany	Croatia

**Population type**	isolated	isolated	isolated	isolated	isolated	general	isolated

**Genotyping platform**	Illumina 318 K	Illumina 318 K	Illumina 318 K	Illumina 318 K	Illumina 318 K	Affymetrix 1000 K	Illumina 370 K

Sample size*	775	1086	653	718	774	1140	895
**Females: n (%)**	472 (61%)	615 (57%)	345 (53%)	385 (54%)	454 (59%)	534 (47%)	572 (64%)
**Age: mean (sd)**	53 (15)	45 (16)	47 (21)	54 (16)	57 (15)	54 (15)	56 (14)
**Diabetes: n (%)**	36 (4.9%)	39 (3.6%)	44 (6.7%)	21 (3.0%)	72 (9.4%)	18 (1.6%)	93 (10.3%)
**AHT^#^: n (%)**	190 (24.5%)	85 (7.8%)	124 (19.0%)	152 (21.2%)	192 (25.2%)	Not available	197 (22.0%)
**S_CR _**mg/dl: **mean (sd)**							
**Males**	1.01 (0.20)	0.96 (0.14)	0.94 (0.20)	0.97 (0.15)	1.01 (0.31)	0.93 (0.15)	0.92 (0.15)
**Females**	0.85 (0.21)	0.78 (0.12)	0.75 (0.14)	0.77 (0.19)	0.79 (0.27)	0.74 (0.12)	0.75 (0.12)
**eGFR^† ^**ml/min/1.73 m^2^							
**Males**	81.1 (19.0)	87.8 (15.1)	92.2 (22.1)	84.3 (16.0)	82.0 (19.4)	88.0 (17.5)	77.1 (14.9)
**Females**	75.8 (20.5)	83.4 (16.8)	88.7 (19.2)	81.5 (18.5)	81.0 (19.8)	85.5 (17.2)	72.4 (14.1)

* Number of individuals with available creatinine, sex, and age information.

^# ^AHT: Anti-hypertensive treatment

^† ^Estimated using the updated abbreviated MDRD study equation [58]

### Replication Cohorts

Data on German healthy control individuals were obtained from the popgen biobank [33]. Genotyping constituted an essential part of the GWAS initiative of the German National Genome Research Network (NGFN) and was performed at an Affymetrix service facility (South San Francisco, CA, USA) using the Affymetrix Genome-Wide Human SNP Array 6.0 (1000 k) (Santa Clara, CA, USA). Genotype calling was carried out using Affymetrix' Birdseed v2 algorithm with default quality thresholds. Samples with more than 5% missing genotypes, showing excess genetic dissimilarity to the remaining subjects, or with evidence for a cryptic relatedness to other study participants were removed. These quality control measures left 1213 control samples for inclusion in the replication cohort. All sex assignments could be verified by reference to the proportion of heterozygous SNPs on the X chromosome. Serum creatinine was available for 1140 individuals and was measured at the Institute for Clinical Chemistry in Kiel, Germany, using an enzymatic *in vitro *assay (CREAplus, Cobas^®^, Roche Diagnostics, Indianapolis, IN).

The *Korcula *study included 944 unselected 18-98 year old Croatians, recruited into the study during 2007 from the island of Korcula [34]. The settlements on Korcula included the Eastern region of the island, which has a unique population history and has maintained a high level of isolation from other mainland populations and from the Western part of the island. Participants were phenotyped for >400 disease-related quantitative traits. Biochemical and physiological measurements were performed, genealogies reconstructed, questionnaires on lifestyle and environmental exposures collected, and blood samples and lymphocytes extracted and stored for further analyses. DNA was genotyped using the Illumina Infinium HumanCNV370v1 SNP bead microarrays. Serum creatinine as measured by the Jaffé rate method was available for 895 individuals.

### Statistical analysis

To ensure normality within centers and comparability of S_CR _values across centers, we applied a quantile normalization in all discovery and replication studies, which involves ranking all S_CR _values and converting them to z-scores according to a standard normal distribution.

#### Discovery stage

To account for inter-individual relatedness within the five EUROSPAN cohorts, genome-wide association (GWA) analysis was carried out following a two stage approach. In the first stage, a sex- and age-adjusted linear model was fitted to the normalized S_CR _in order to estimate the residuals. A polygenic model was then fitted to estimate the inverse of the variance-covariance matrix, which accounts for the inter-individual relatedness and is based upon a genomic kinship matrix as described in Amin *et al*. [35]. The association between SNPs and residuals was assessed by means of an approximate score test statistic [36], assuming an additive model, as implemented in the GenABEL package [37]. The results from the five EUROSPAN cohorts were then combined into a fixed-effects meta-analysis with inverse-variance weighting, using MetABEL http://mga.bionet.nsc.ru/~yurii/ABEL/. Only SNPs that had a call rate ≥ 0.95, a Hardy-Weinberg equilibrium (HWE) test p value > 10^-6 ^and a minor allele frequency (MAF) ≥ 0.01 were included in the analyses. In total, 322,498 SNPs were tested for association with S_CR_. The threshold for genome-wide statistical significance, according to Bonferroni adjustment for multiple testing, was set to 1.55 × 10^-7^. Between-study heterogeneity was quantified using the I^2 ^statistic, i.e. the percentage of total variation explained by heterogeneity rather than sampling error [38].

#### Replication stage

**Figure 1:**
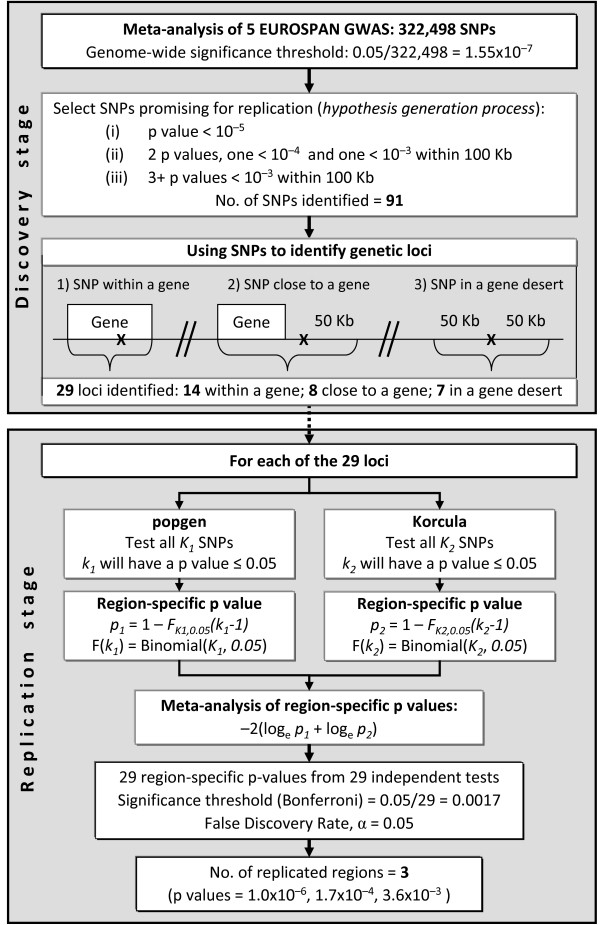
**Workflow of the discovery and replication stages of the study (for details, see the *Statistical Analysis *section)**.

In the absence of clear methodological guidance on what may be the best strategy for passing SNPs to a replication stage, we selected SNPs for replication based on a tradeoff that enabled us to include our best findings from the discovery analysis, whilst avoiding the risk of an excessively long list. This was achieved by including all SNPs with a p value ≤ 10^-5^, but also allowing for SNPs with higher p values to get into the replication list in the presence of additional evidence for association provided by other SNPs within 100 kb. The following criteria were applied: (i) at least one p value ≤ 10^-5^; (ii) at least one p value ≤ 10^-4 ^and at least one additional SNP with p value ≤ 10^-3 ^within 100 kb; (iii) at least three SNPs with p value ≤ 10^-3 ^within 100 kb. For each group of candidate SNPs, we selected a candidate gene (all SNPs included in the gene were considered for replication) or region (all SNPs included within ± 100 kb of the candidate SNPs were considered for replication) to test in the independent cohorts of Korcula and popgen. The complete workflow of the test procedure is depicted in Figure 1.

In popgen, the association between S_CR _and each SNP within a candidate region was assessed by means of a sex- and age-adjusted linear regression model assuming additive genetic effects, using PLINK version 1.05 http://pngu.mgh.harvard.edu/purcell/plink/[39]. For Korcula, given that it is a family-based study, we used the same approach as described above for the EUROSPAN cohorts.

To assess the significance in the replication cohorts of associations with different SNPs from those genotyped in the discovery cohorts, we defined region-specific p values as follows: for a genomic region containing *N *SNPs, we counted the number *n *of SNPs that achieved p value < 0.05. Assuming that p values are uniformly distributed between 0 and 1 under the null hypothesis of no association, the distribution function of *n*, *F(.)*, is that of a binomial with parameters *N *and 0.05. The region-specific p value then equals *1-F*_*N,0.05*_*(n-1)*. Given that in small genomic regions the assumption of independency is rarely met, the estimated p value should be considered as conservative since the effective number of independent tests can only be lower than the total number of SNPs tested. Meta-analysis of region-specific p values from popgen and Korcula was finally performed using the Fisher's combined probability test [40], which is suitable for combining tests performed in independent samples. We further evaluated rejection of the null hypothesis of no association based on a false discovery rate (FDR) of 0.05 [41], where p values are sorted in ascending order and the first *k *tests with p value ≤ *i/m *× *α *are considered significant (in our case, *i = 1..29*, *m = 29*, *α = 0.05)*.

#### Replication of previous findings

We finally assessed whether any of the four loci reported to be associated with eGFR by Köttgen *et al*. [18] showed evidence for an association with S_CR _in our discovery meta-analysis. Köttgen *et al*. reported four SNPs to be associated with GFR: rs17319721 (*SHROOM3*), rs2467853 (*SPATA5L1-GATM*), rs12917707 (*UMOD*), and rs6040055 (*JAG1*). For each of the four SNPs, we defined LD blocks around them, following the method by Gabriel *et al*. [42] and using SNPs with MAF ≥ 1% from the HapMap-CEU database, Phase III/Release 2. Given that the genes were in four independent loci, the threshold for claiming significant replication, according to Bonferroni, was set to 0.05/4 = 0.0125. To compare our results with the commonly used method of testing replication at the same SNP or at a proxy SNP, we repeated the test procedure at one SNP per locus, using the same threshold for statistical significance.

If not specified otherwise, all data management, data analysis, programming, and the creation of graphs were performed using R 2.8.0 [43].

## Results

Study-specific characteristics of the participants are reported in Table 1. A total of 4006 individuals were included in the meta-analysis whilst the replication cohorts comprised 2035 individuals. All EUROSPAN cohorts comprised a higher percentage of females (between 53% and 61%) than males. The mean age ranged from 45 years in MICROS to 57 years in VIS. The replication samples had a similar age range, but a smaller percentage of females than males included in popgen.

**Figure 2:**
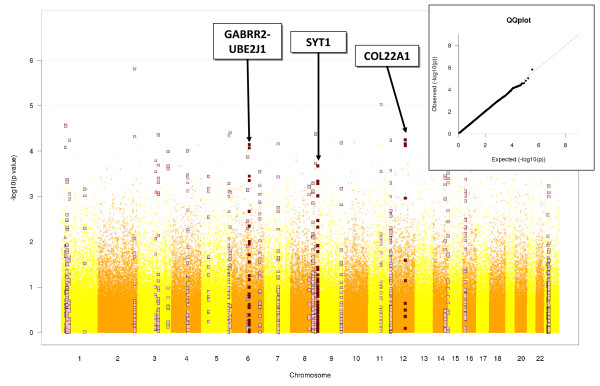
**Manhattan plot of -log_10 _(p values) from the meta-analysis of five GWA studies**. GWA results were combined across all studies by fixed-effects meta-analysis using inverse variance weighting. Square dots indicate SNPs satisfying criteria for selection of candidate regions. SNPs within replicated regions are colored in dark red and SNPs within non-replicated regions in pink. The genomic control factor of λ = 1.004, assessed by the quantile-quantile plot in the upper-right panel, indicates that cryptic relatedness and population structure have been modeled appropriately.

The results of the GWA meta-analysis are depicted in Figure 2. The genomic control factor (λ), as assessed by the quantile-quantile plot included in Figure 2, was 1.004 (SE < 10^-5^), indicating that no cryptic relatedness or gross population structure affected our results [44].

The smallest p value in the meta-analysis was 1.5 × 10^-6 ^and was obtained for SNP rs2396463 in the collagen type IV alpha-3 (*COL4A3*) gene on chromosome 2q. In the absence of hits of genome-wide significance, we selected a set of promising regions for follow-up in two independent replication samples, namely Korcula (an island population from Croatia) and popgen (a random population sample from the most northern part of Germany). Twenty-nine genomic regions were considered for replication. The full list of regions and the results of the discovery meta-analysis are provided in the Additional file 1 - Table S1. Since the SNP panels genotyped in the replication samples differed from one another and from that used in the discovery GWA studies, all markers present in the candidate regions were included in the replication phase. A total of 2224 SNPs were analyzed in the popgen samples and 1136 SNPs were analyzed in the Korcula samples. Results of the replication analysis are fully reported in the Additional file 1 - Table S2, with loci sorted by significance.

**Figure 3:**
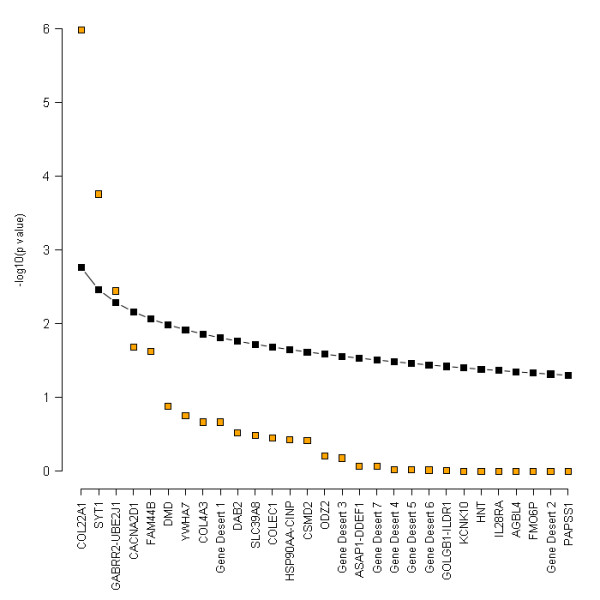
**Results of the false discovery rate analysis**. -log_10 _(Fisher p value) of the replication analysis (orange) and -log_10 _(significance thresholds) for FDR (black) are plotted for each candidate locus. According to [41], FDR thresholds have been calculated as *i/m × α*, with *α *= 0.05, *m *= no. of test (i.e.: no. of regions, 29), and *i *ranging between 1 and 29. After sorting the Fisher p values in ascending order, the first *k *tests for which the p value is ≤ threshold are significant.

Two candidate regions from the discovery GWA studies also showed significant evidence for an association with S_CR _level in the popgen and Korcula samples after Bonferroni correction for multiple testing of the combined p value (significance threshold for 29 independent tests: α = 0.0017). When controlling for the FDR, i.e. for the probability of reporting a false positive result, a third region could be added to the list of replicated findings (Figure 3).

**Figure 4:**
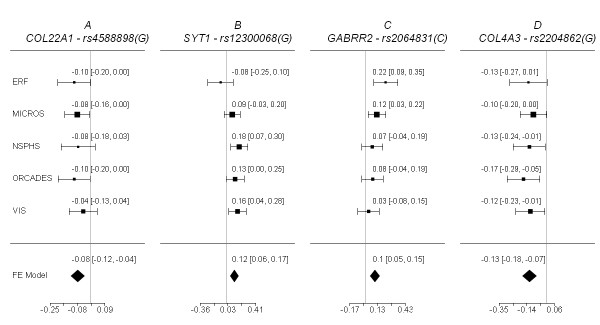
**Forest plots of betas and standard errors, with 95% confidence intervals, for the most significant SNPs in the three replicated loci (A, B, and C) and for the most significant SNP of the discovery meta-analysis (D)**. The pooled estimate was obtained with a fixed-effect meta-analysis using the *metafor *package in R http://www.wvbauer.com/index.htm.

**Figure 5:**
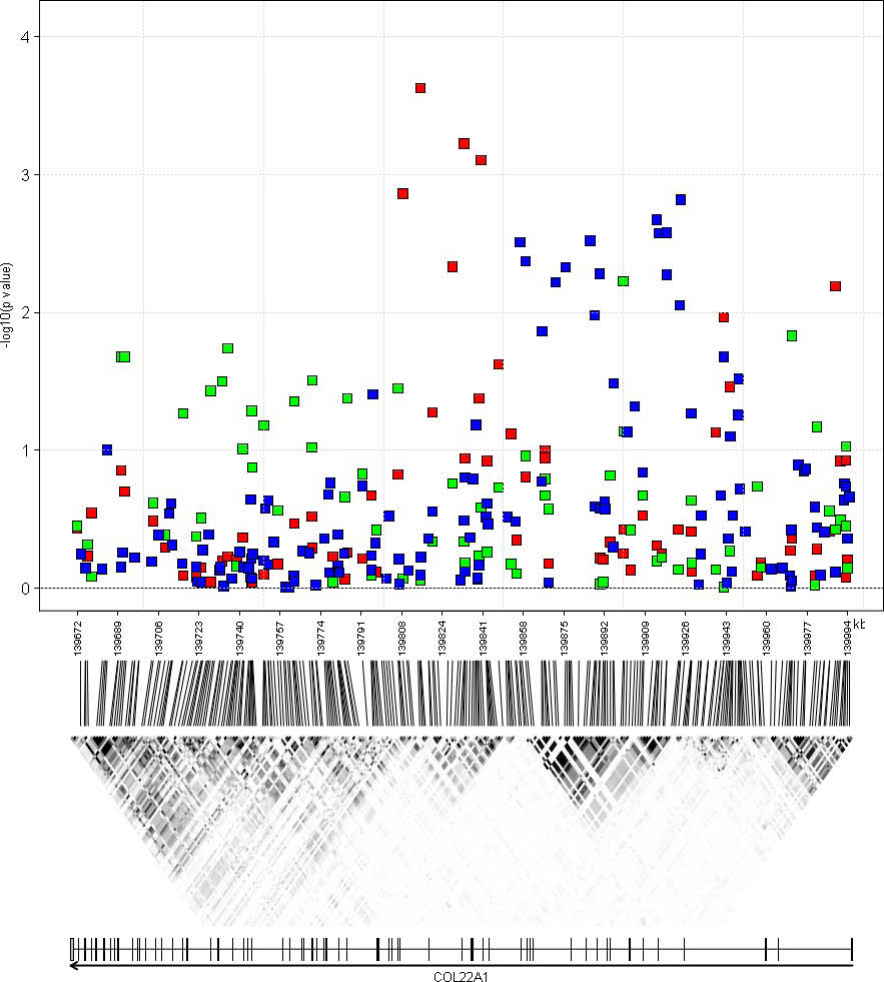
**Genomic structure and association results at the *COL22A1 *locus**. **Upper panel**: -log_10_(p values) are plotted by physical position for the EUROSPAN discovery meta-analysis (red squares), the popgen (blue squares) and the Korcula (green squares) replication cohorts. **Middle panel**: linkage disequilibrium (LD) as quantified by r^2 ^(the higher the LD, the darker the color, with black indicating perfect LD), based upon the HapMap-CEU database, Phase III/Release 2, (NCBI build 36). **Lower panel**: genes located in the plotted region, with coding exons indicated by black rectangles and orientation.

The first replicated region was defined by four SNPs (rs4075073, rs4588898, rs9324496, and rs2873682) in the collagen type XXII alpha 1 (*COL22A1*) gene, all of which yielded p values ≤ 9.79 × 10^-4 ^in the meta-analysis of the discovery stage, with homogeneous effects observed across populations: I^2 ^= 0% for all the four SNPs. A forest plot of the marker with the strongest evidence for association, SNP rs4588898, is provided in Figure 4A. Six other SNPs in the gene (out of 72) had p values between 3.42 × 10^-3 ^and 0.0414 (Table 2). As it can be inferred from Figure 5, some 19 of 140 *COL22A1 *SNPs genotyped in popgen were found to be significantly associated with S_CR _(red squares), as were 11 out of 71 SNPs in Korcula (green squares). Fisher's combined probability test p value was 1.0 × 10^-6^. Nevertheless, the gene regions with the strongest S_CR _association in EUROSPAN and either popgen or Korcula failed to overlap and were found to be located in distinct LD blocks. Interestingly, however, the smallest p values for popgen and Korcula fell into the same LD block.

**Table 2:** SNPs showing the highest evidence of association in the *COL22A1 *gene (chromosome 8).^#^

study	name	position	Genotype (Ref. All.)	**Ref. All. Freq**.	n	Beta*	SE	p value**
EUROSPAN	rs4075073	139808143	A/C (A)	0.37	3996	0.0671	0.0203	9.8 × 10^-4^
EUROSPAN	rs4588898	139815390	A/G (A)	0.30	3999	0.0786	0.0212	2.1 × 10^-4^
EUROSPAN	rs13255079	139828786	C/T (T)	0.16	4001	0.0769	0.0263	3.4 × 10^-3^
EUROSPAN	rs9324496	139833578	C/T (T)	0.34	3995	0.0716	0.0205	4.6 × 10^-4^
EUROSPAN	rs7824025	139839898	A/C (A)	0.07	3998	0.0762	0.0374	0.0414
EUROSPAN	rs2873682	139840693	A/G (A)	0.30	2610	0.0915	0.0264	5.2 × 10^-4^
EUROSPAN	rs11166844	139848062	C/T (C)	0.39	3993	0.0485	0.0202	0.0166
EUROSPAN	rs1574172	139941802	A/G (A)	0.14	3998	-0.0713	0.0284	0.0122
EUROSPAN	rs4736078	139944298	C/T (C)	0.22	3996	-0.0503	0.0242	0.0373
EUROSPAN	rs6986041	139988449	C/T (C)	0.35	3936	-0.0583	0.0207	4.8 × 10^-3^
								
POPGEN	rs4282617	139795530	C/G (C)	0.25	1139	-0.0822	0.0398	0.0391
POPGEN	rs10092896	139856976	C/T (C)	0.27	1139	-0.1123	0.0380	3.1 × 10^-3^
POPGEN	rs2318334	139859320	C/T (C)	0.25	1140	-0.1115	0.0390	4.2 × 10^-3^
POPGEN	rs1574370	139866164	A/G (A)	0.24	1126	-0.0992	0.0402	0.0137
POPGEN	rs6991720	139871695	C/G (G)	0.25	1139	-0.1074	0.0391	6.1 × 10^-3^
POPGEN	rs7837787	139875897	A/G (G)	0.25	1140	-0.1106	0.0391	4.7 × 10^-3^
POPGEN	rs12155960	139886087	A/G (G)	0.26	1134	-0.1152	0.0388	3.0 × 10^-3^
POPGEN	rs11779129	139888085	A/C (A)	0.25	1140	-0.0992	0.0388	0.0105
POPGEN	rs16893541	139890024	C/G (C)	0.27	1124	-0.1089	0.0390	5.2 × 10^-3^
POPGEN	rs12549194	139896019	A/C (C)	0.26	1137	-0.0830	0.0388	0.0327
POPGEN	rs10093925	139904787	C/T (T)	0.36	1124	-0.0687	0.0347	0.0478
POPGEN	rs6577953	139914021	C/G (C)	0.40	1139	-0.1032	0.0336	2.1 × 10^-3^
POPGEN	rs6993839	139914702	A/G (G)	0.39	1131	-0.1029	0.0342	2.6 × 10^-3^
POPGEN	rs9650564	139918011	C/T (C)	0.41	1139	-0.1017	0.0338	2.6 × 10^-3^
POPGEN	rs9650565	139918089	C/T (C)	0.41	1133	-0.0944	0.0339	5.3 × 10^-3^
POPGEN	rs7014497	139923457	C/T (C)	0.39	1133	-0.0892	0.0341	8.9 × 10^-3^
POPGEN	rs9650566	139923872	A/G (G)	0.46	1139	-0.1054	0.0332	1.5 × 10^-3^
POPGEN	rs17740495	139941879	C/T (C)	0.08	1136	0.1393	0.0603	0.0209
POPGEN	rs16893545	139948143	A/C (C)	0.18	1140	0.0953	0.0440	0.0304
								
KORCULA	rs4076439	139690550	A/G (G)	0.25	895	0.0907	0.0393	0.0209
KORCULA	rs4341165	139692067	C/T (C)	0.25	895	0.0907	0.0393	0.0209
KORCULA	rs4909439	139727929	C/T (C)	0.17	895	0.1080	0.0518	0.0371
KORCULA	rs3923549	139732799	C/T (T)	0.10	895	0.1513	0.0704	0.0316
KORCULA	rs4073446	139735056	C/T (T)	0.11	895	0.1555	0.0658	0.0182
KORCULA	rs7839680	139762863	A/G (A)	0.18	895	0.0950	0.0472	0.0440
KORCULA	rs4909444	139770391	G/T (T)	0.38	895	0.0623	0.0289	0.0309
KORCULA	rs4074052	139785008	C/T (T)	0.46	894	-0.0504	0.0248	0.0419
KORCULA	rs10112806	139806115	A/C (C)	0.41	895	-0.0589	0.0280	0.0357
KORCULA	rs1320279	139899978	C/T (C)	0.11	895	-0.1823	0.0662	5.9 × 10^-3^
KORCULA	rs2318345	139970360	C/T (C)	0.09	894	0.1755	0.0720	0.0147

^# ^association models were adjusted for age and sex; *in standard deviations; **p value: for EUROSPAN, the p value refers to the meta-analysis of the five individual GWA studies, for popgen and Korcula, the p value is from the linear regression analysis (for details, see Methods).

Three closely linked SNPs (rs10506807, rs12300068, and rs11112829) on chromosome 12q21, yielded p values ≤ 7.55 × 10^-5 ^in the EUROSPAN discovery meta-analysis. I^2 ^for heterogeneity was 21.88%, 24.11%, and 19.94%, respectively, none of which was statistically significant. A forest plot for SNP rs12300068 is provided in Figure 4B. Depending upon the annotation of the 5'-UTR sequence, all SNPs are located either >100 kb upstream (NCBI) or directly inside (Ensembl and UCSC) the synaptotagmin-1 (*SYT1*) gene. In the popgen sample, nine SNPs located less than 50 kb upstream of the three discovery SNPs were significantly associated with S_CR_, with p values between 2.0 × 10^-5 ^and 8.5 × 10^-3 ^(Table 3). The Fisher's combined probability test p value for the whole region was as low as 1.7 × 10^-4^.

**Table 3:** SNPs with the highest evidence of association in the region upstream the *SYT1 *gene.^#^

Study	name	position	Genotype (Ref. All.)	**Ref. All. Freq**.	n	Beta*	SE	p value**
EUROSPAN	rs11838060	77997790	G/T (T)	0.14	3163	-0.0696	0.0312	0.0258
EUROSPAN	rs10506807	78005428	C/T (C)	0.13	4000	-0.1141	0.0287	7.0 × 10^-5^
EUROSPAN	rs12300068	78005502	A/G (A)	0.13	3997	-0.1158	0.0288	5.7 × 10^-5^
EUROSPAN	rs7972593	78013149	C/T (T)	0.12	3989	-0.0978	0.0300	1.1 × 10^-3^
EUROSPAN	rs11112829	78029267	C/T (C)	0.13	4000	-0.1135	0.0287	7.5 × 10^-5^
								
POPGEN	rs12312807	77957336	A/G (A)	0.11	1139	0.1480	0.0539	6.1 × 10^-3^
POPGEN	rs1527119	77964145	G/T (G)	0.11	1139	0.1471	0.0539	6.4 × 10^-3^
POPGEN	rs1033196	77966968	C/T (T)	0.14	1128	0.1320	0.0479	5.8 × 10^-3^
POPGEN	rs2950383	77973538	A/G (A)	0.37	1103	-0.1553	0.0359	2.0 × 10^-5^
POPGEN	rs1356022	77974533	C/T (C)	0.14	1140	0.1300	0.0475	6.2 × 10^-3^
POPGEN	rs12317960	77977824	C/T (T)	0.14	1140	0.1249	0.0474	8.5 × 10^-3^
POPGEN	rs10506806	77979113	C/T (T)	0.31	1120	0.1012	0.0362	5.1 × 10^-3^
POPGEN	rs11610381	77979918	C/T (C)	0.14	1134	0.1321	0.0476	5.5 × 10^-3^
POPGEN	rs7971081	77980051	C/T (C)	0.14	1140	0.1296	0.0474	6.3 × 10^-3^

^# ^association models were adjusted for age and sex; *in standard deviations; **p value: for EUROSPAN, the p value refers to the meta-analysis of the five individual GWA studies, for popgen, the p value is from the linear regression analysis (for details, see Methods).

**Figure 6:**
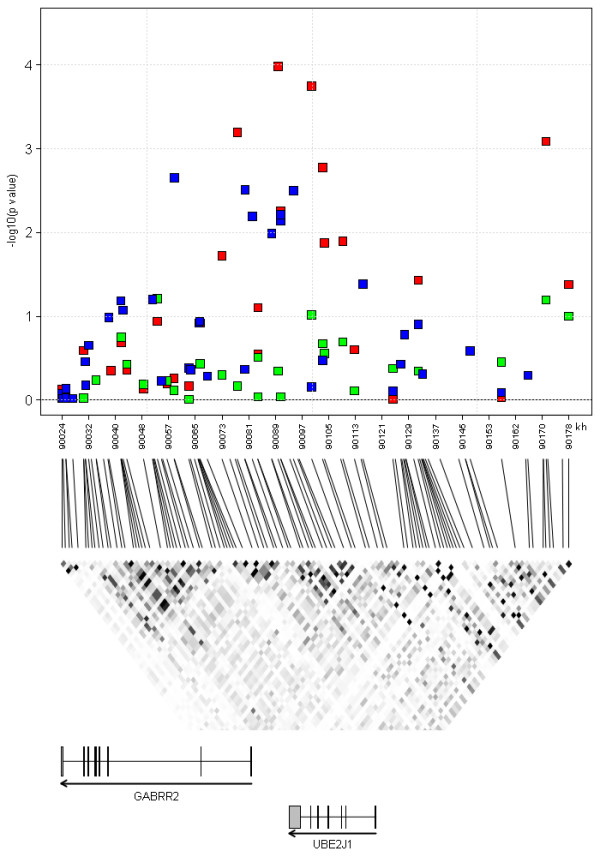
**Genomic structure and association results at the *GABRR2-UBE2J1 *locus**. **Upper panel**: -log_10_(p values) are plotted by physical position for the EUROSPAN discovery meta-analysis (red squares), the popgen (blue squares) and the Korcula (green squares) replication cohorts. **Middle panel**: linkage disequilibrium (LD) as quantified by r^2 ^(the higher the LD, the darker the color, with black indicating perfect LD), based upon the HapMap-CEU database, Phase III/Release 2, (NCBI build 36). **Lower panel**: genes located in the plotted region, with coding exons indicated by black rectangles and orientation.

The third locus was defined by the four SNPs rs3777514 (p value = 4.5 × 10^-4^), rs2064831 (p value = 7.2 × 10^-5^), rs1998576 (p value = 8.6 × 10^-5^), and rs7744005 (p value = 3.6 × 10^-4^). The effect of the three SNPs upon S_CR _level was homogeneous across populations, with an I^2 ^of 2.20% (p value = 0.38), 6.73% (p value = 0.29), 9.70% (p value = 0.38), and 0.00% (p value = 0.54), respectively. A forest plot of the SNP with the strongest evidence for association (rs2064831), is reported in Figure 4C. The SNPs in question are located in a region on chromosome 6q15 containing the gamma-aminobutyric acid receptor rho-2 (*GABRR2*) and the ubiquitin-conjugating enzyme E2-J1 (*UBE2J1*) genes (Figure 6). Five additional SNPs (out of a total of 28 SNPs tested) in this region had p values between 2.2 × 10^-3 ^and 0.0280 (Table 4). Of the 37 SNPs genotyped in the popgen sample, eight were associated with S_CR _with a p value ≤ 0.05; seven of them overlapped with the *GABRR2 *and *UBE2J1 *genes (see the blue squares in Figure 6). The most consistent association signals in EUROSPAN and popgen were found near the *GABRR2 *gene and its promoter, and the respective SNPs were in linkage disequilibrium (r^2 ^> 0.59, Figure 6, middle panel). SNP rs12195070 was genotyped in both EUROSPAN and popgen: effect direction was discordant but in both cases association was very significant with p values of 4.5 × 10^-3 ^in EUROSPAN and p value = 7.2 × 10^-3 ^in popgen, respectively. In the Korcula samples, the smallest p value in the region was 0.0616 so that we cannot formally claim replication in this population. However, Fisher's combined probability test p value equaled 3.6 × 10^-3^. This value does not meet the Bonferroni threshold for multiple testing, but it does meet the FDR threshold of 0.0052, which controls the probability of reporting false positives at a 0.05 level (see Additional file 1 - Table S2 and Figure 3). Detailed results for this locus are reported in Table 4.

**Table 4:** SNPs with the highest evidence of association in the region including the *GABRR2 *and the *UBE2J1 *genes (chromosome 6)^#^.

Study	GENE	name	Position	Genotype (Ref. All.)	**Ref. All. Freq**.	N	Beta*	SE	p value**
EUROSPAN	GABRR2	rs6942204	90072687	C/T (C)	0.34	4001	0.0497	0.0213	0.0196
EUROSPAN	GABRR2	rs3777514	90077300	A/C (A)	0.20	3998	0.0855	0.0244	4.5 × 10^-4^
EUROSPAN	Intergenic	rs2064831	90089661	C/T (C)	0.18	3995	0.1018	0.0257	7.2 × 10^-5^
EUROSPAN	Intergenic	rs12195070	90090455	C/T (T)	0.19	3998	0.0722	0.0254	4.5 × 10^-3^
EUROSPAN	UBE2J1	rs1998576	90099856	A/G (A)	0.18	3997	0.0993	0.0253	8.6 × 10^-5^
EUROSPAN	UBE2J1	rs12189673	90103123	C/T (C)	0.30	3980	-0.0653	0.0213	2.2 × 10^-3^
EUROSPAN	UBE2J1	rs1065657	90103732	C/T (T)	0.45	3999	0.0499	0.0197	0.0115
EUROSPAN	UBE2J1	rs7760851	90109323	A/G (G)	0.49	3980	-0.0505	0.0196	0.0100
EUROSPAN	UBE2J1	rs1062108	90132078	C/T (T)	0.42	3996	-0.0435	0.0198	0.0280
									
POPGEN	GABRR2	rs9362633	90058261	A/G (G)	0.05	1136	-0.2430	0.0795	2.2 × 10^-3^
POPGEN	GABRR2	rs7764923	90079640	C/T (T)	0.23	1140	-0.1144	0.0387	3.1 × 10^-3^
POPGEN	GABRR2	rs2236204	90081831	G/T (T)	0.17	1121	-0.1190	0.0436	6.4 × 10^-3^
POPGEN	Intergenic	rs10944443	90087709	C/G (C)	0.15	1113	-0.1203	0.0469	0.0103
POPGEN	Intergenic	rs12195070	90090455	C/T (T)	0.18	1140	-0.1155	0.0430	7.2 × 10^-3^
POPGEN	Intergenic	rs12195078	90090511	C/T (T)	0.15	1128	-0.1261	0.0460	6.1 × 10^-3^
POPGEN	UBE2J1	rs9048	90094300	C/T (T)	0.18	1139	-0.1230	0.0417	3.1 × 10^-3^
POPGEN	UBE2J1	rs9351207	90115388	C/T (T)	0.24	1131	-0.0791	0.0387	0.0412

^# ^association models were adjusted for age and sex; *in standard deviations; **p value: for EUROSPAN, the p value refers to the meta-analysis of the five individual GWA studies, for popgen, the p value is from the linear regression analysis (for details, see Methods).

Although consistently observed in all EUROSPAN populations (Figure 4D), the association noted between S_CR _and the *COL4A3 *gene could not be verified in the replication cohorts (Fisher's combined probability test p value = 0.216).

Unfortunately, the number of people with diabetes included in the EUROSPAN populations was too small to allow any meaningful subgroup analysis, adjusting for disease state. However, when we repeated our GWA meta-analysis in non-diabetic subjects only, the p values for the three replicated gene regions changed so little that any strong interaction of the respective loci with diabetes status appears rather unlikely.

Results of the replication analysis at the four loci reported to be associated with eGFR by Köttgen *et al*. [18] are reported in the Additional file 1 - Table S3. We identified three SNPs in the *UMOD *gene locus: rs4293393 was significantly associated with S_CR _level (p value = 3.9 × 10^-4^) and weaker signals were observed for rs11647727 (p value = 0.011) and rs4506906 (p value = 0.028). The region-specific p value for this locus was 1.3 × 10^-4^. Of the five SNPs identified in the *SPATA5L1-GATM *locus, rs1153860, rs1346268, and rs1719247 were associated with S_CR _at p values of 5.0 × 10^-4^, 1.9 × 10^-3^, and 4.6 × 10^-3^, respectively (region-specific p value = 1.2 × 10^-3^). At the *SCHROOM3 *gene locus, 53 SNPs were tested: three of them resulted in a p value ≤ 0.05 (region-specific p value = 0.50). The smallest p value of 1.7 × 10^-3 ^was observed for SNP rs4859682 (this SNP was in strong LD with the rs17319721 reported in the paper, r^2 ^= 0.94). None of the 8 polymorphisms tested in the *JAG1 *gene was significantly associated with S_CR _level, including the rs6040055 which was included in our database as well. Our conclusions would have been slightly different had we attempted replication at the level of the individual variants rather than at the locus level. In this case, also the SCHROOM3 locus would have been replicated.

## Discussion

The main result of our study was the identification of three novel genetic associations with S_CR _level. These were detected in a meta-analysis of five European isolated population samples and replicated in two independent population samples, one from the general Central-European population (popgen), and one from another isolate (Korcula). The replicated loci included the *COL22A1 *gene on chromosome 8 and the *SYT1 *gene on chromosome 12. The third locus, including the *GABRR2 *and *UBE2J1 *genes on chromosome 6, showed substantial evidence of association as well. In addition, the association of two loci (*UMOD *and *SPATA5L1-GATM*) and one SNP in the *SCHROOM3 *locus, previously reported with GFR, was also found to apply to S_CR_. This confirmation also supports our choice of using S_CR _adjusted by age and sex which is equivalent to study GFR calculated from S_CR _weighted by age and sex [1] (black or white ethnicity, which should also be accounted for, was not relevant in our study involving individuals of European origin).

The two SNPs with the strongest evidence for association in the discovery stage of our study were located in the promoter region of the *COL4A3 *gene, which is expressed in the glomerular basement membrane (GBM) of the kidney, and which has been associated with Alport syndrome, Goodpasture syndrome [45] and benign hematuria [46]. Despite making biological sense, however, the association between *COL4A3 *and S_CR _as observed in our meta-analysis could not be verified in the replication stage. On the one hand, this could be interpreted to indicate that no common variants of that gene are involved in the regulation of kidney function. On the other hand, the lack of replication could be due either to the involvement of the gene in regulatory pathways, implying a strong interaction with other genes, or to a particularly prominent interaction with population-specific environmental conditions [47].

In any case, a slightly less significant association between three other genes and S_CR _from the discovery meta-analysis could be replicated in either the popgen or the Korcula data. Different SNPs in the *COL22A1 *gene were found to be associated with S_CR _in all discovery and replication cohorts. This association may reflect the biological relationship between muscle mass formation and creatinine levels. In fact, *in situ *hybridization of myotendinous junctions has revealed that muscle cells produce collagen XXII, and functional tests have shown that collagen XXII acts as a cell adhesion ligand for skin epithelial cells and fibroblasts [48].

The discovery stage association of S_CR _with the *SYT1 *gene was well replicated in the popgen sample. Even though the annotation of gene reference sequences is not uniform across different databases (NCBI, UCSC, Ensembl), the associated SNPs are located in the fourth and last intron of the 5' *SYT1 *un-translated region (UTR), suggesting a regulatory role for this locus. Data on the clones available from public databases clearly show that *SYT1 *transcription starts around 77,782 kb and 77,963 kb on chromosome 12 and that the transcripts are spliced in the 5'UTR region (see, among others, the mRNA clones with GI numbers 37589129, 21753708, 34533586, 34366268, 37589129, 164696663). The synaptotagmins are integral membrane proteins of synaptic vesicles thought to serve as Ca(2+) sensors in the process of vesicular trafficking and exocytosis [49]. Of particular interest in the present context is expression of the *SYT1 *gene product, synaptotagmin-I, in renal podocytes. Glomerular podocytes possess structures resembling synaptic vesicles and contain glutamate, they co-express *Rab3A *(a GTPase restricted to cell types that regulate exocytosis), synapsin, synaptophysin, and synaptotagmin-I, and undergo spontaneous and stimulated exocytosis and recycling, with glutamate release [50]. Synaptotagmin-I plays a role in neurotransmitter release and neurite outgrowth [51] and is thought to interact with neurotransmitters such as the GABA receptors, expressed in the GBM. It also appears to participate in the regulation of podocyte homeostasis [50].

In light of the findings on the *SYT1 *gene, the association with the *UBE2J1 *and *GABRR2 *locus becomes of high interest. The association detected in the EUROSPAN data was corroborated by the popgen data. Moreover, SNPs associated with S_CR _in popgen enabled us to narrow down the associated region to the *GABRR2 *promoter. The same locus was not replicated in Korcula data. However, while the popgen genotype distribution of the SNPs in question was found to resemble that of the HapMap-CEU samples [52], indicating that popgen can be considered representative of the general Central to Northern European populations, this may not be true for the Korcula sample. In fact, Korcula is a small population located in the Croatian islands, where pronounced genetic isolation is known to have persisted until recently [53]. This could be the reason of the missed replication. The *GABRR2 *gene product is a member of the rho subunit family of the transmembrane receptors for gamma-aminobutyric acid (GABA_A_), which are linked to potassium channels via G-proteins. GABA_A _receptors have been shown to be expressed in the kidney of multiple species, with subunits β_1 _and β_2 _localized in the proximal tubules [54]. More recently, several subtypes of GABA receptors, including the *GABRR2*, have been shown to be transcribed in normal human glomeruli [50] and it plays a role in retinal neurotransmission [55]. The gene is located close to the *GABRR1 *gene in the same transcriptional orientation, suggesting a similar expression and regulatory pattern. Interestingly, the locus falls into the supporting interval for linkage to S_CR _reported in Mexican Americans [7], and to creatinine clearance as reported in Caucasians [9].

Our discovery analysis was performed in five isolated communities from Southern, Central, and Northern Europe. As has been demonstrated empirically for South Tyrolean villages [56], population isolates provide a reduced within-study heterogeneity of environmental factors, thereby facilitating the genetic dissection of complex traits. This internal homogeneity of environmental and life style factors could be counterbalanced by enhanced between-study heterogeneity. However, this appears not to have been the case in our study. For SNPs in 27 of the 29 regions selected for replication, the hypothesis of homogeneity of effects' sizes was not rejected, even at a very stringent significance threshold (given the small number of studies involved in the meta-analysis, α was set to 0.10). In particular, homogeneity of association signals was verified for all replicated loci and for the *COL4A3 *gene locus. This finding strengthens our results in the sense that, despite the expected between-population heterogeneity, the results of the association analyses were found to be very consistent across studies.

Another advantage of our study has been that S_CR _values of all EUROSPAN participants were measured in one and the same centralized laboratory using the enzymatic method. In this way, calibration difference could be excluded as a confounder of our genetic association analyses. While the enzymatic method was used to measure S_CR _in EUROSPAN and popgen participants, the Jaffé rate method was used in the Korcula study. This method is known to be less precise than the enzymatic one. Additional noise introduced by increased measurement error would best explain the reduced evidence for association replication in the Korcula study. However, S_CR _was standardized in all discovery and replication samples using a transformation based upon the ranks of a standard normal distribution. The standardization of the phenotype measures allowed us to combine data across studies avoiding bias due to technical inter-laboratory differences.

The validity of our analysis was also evidenced by the replication of earlier association findings [18]. Association of S_CR _with *UMOD *and *SPATA5L1-GATM *was confirmed using the same replication approach used in our own analysis. With our method, however, we could not replicate the association over the *SCHROOM3 *locus (the locus would be confirmed if using a classic replication based on the specific SNP or a proxy SNP). This finding could be an indication that our method of replication is more likely to be conservative and, so, less prone to false positive results. In the *COL22A1 *and the *SYT1 *genes the replicated signals were located in different LD blocks, highlighting the possibility that different polymorphisms at the same locus may be associated with the same phenotype in different populations. In the *GABRR2 *- *UBE2J1 *gene locus, association signals between discovery and replication studies were discordant but clearly overlapping. Whether the discordance of the effect alleles is an indication of a false positive result is very unlikely, given the size of the effects in EUROSPAN and in popgen. The presence of a flip-flop phenomenon [57] could be hypothesized and should be investigated further.

## Conclusions

In conclusion, in addition to confirming earlier findings, our search for genes associated with S_CR _variation led to the discovery of three novel genes that represent sensible candidates for further functional analysis. The recent hypothesis of an important role of neurotransmitters in the regulation of the GBM at the podocyte level [50] renders our findings particularly relevant for understanding the regulation of renal function, suggesting that the possible interaction between SYT1 and *GABRR2 *warrants further investigation.

## Competing interests

The authors declare that they have no competing interests. The funders had no role in the study design, the data collection and analysis, the decision to publish, or the preparation of the manuscript.

## Authors' contributions

SHW, OP, UN, SS, NH, TM, BO, AFW, HC, CMvD, UG, JFW, MK, IR, and PPP conceived and designed the studies. CP, VV, CH, YSA, AJ, AI, DE, and AF performed the data analysis. CP, ADG, SAM, PR, CM, and MK discussed and provided interpretation of the results. CP, ADG, VV, AF, SHW, IK, LZ, TZ, CG, SS, SC, MB, PR, CM, HC, UG, and MK wrote and revised the manuscript. All authors gave final approval of the manuscript to be published.

## Pre-publication history

The pre-publication history for this paper can be accessed here:

http://www.biomedcentral.com/1471-2350/11/41/prepub

## Supplementary Material

Additional file 1 **Supplementary materials**. The file contains supplementary tables to support the discovery analysis (Table S1), the replication analysis (Table S2) and the confirmation of previous findings (Table S3). Click here for file
