# Supplementary material for: A meta-analysis of genome-wide data from five European isolates reveals an association of COL22A1, SYT1, and GABRR2 with serum creatinine level
Source: BMC Med Genet. 2010 Mar 11;11:41. doi: 10.1186/1471-2350-11-41 (PMC2848223; doi:10.1186/1471-2350-11-41)
Supplement: Additional file 1 — Supplementary materials. The file contains supplementary tables to support the discovery analysis (Table S1), the replication analysis (Table S2) and the confirmation of previous findings (Table S3). [file 1471-2350-11-41-S1.DOC]

**Supplementary Materials**

**A meta-analysis of genome-wide data from five European isolates reveals an association of *COL22A1*, *SYT1*, and *GABRR2* with serum creatinine level.**

Cristian Pattaro^1*^, Alessandro De Grandi^1^, Veronique Vitart^2^, Caroline Hayward^2^, Andre Franke^3^, Yurii S Aulchenko^4^, Asa Johansson^5^, Sarah H Wild^6^, Scott A Melville^1^, Aaron Isaacs^4^, Ozren Polasek^7,8^, David Ellinghaus^3^, Ivana Kolcic^7^, Ute Nöthlings^9,10^, Lina Zgaga^7^, Tatijana Zemunik^11^, Carsten Gnewuch^12^, Stefan Schreiber^3^, Susan Campbell^2^, Nick Hastie^2^, Mladen Boban^11^, Thomas Meitinger^13,14^, Ben A Oostra^4^, Peter Riegler^15^, Cosetta Minelli^1^, Alan F Wright^2^, Harry Campbell^6^, Cornelia M van Duijn^4^, Ulf Gyllensten^5^, James F Wilson^6^, Michael Krawczak^9,16^, Igor Rudan^6,8,11^, Peter P Pramstaller^1,17,18*^, on behalf of the EUROSPAN consortium

^1^ Institute of Genetic Medicine, European Academy Bozen/Bolzano (EURAC), Bolzano, Italy - Affiliated Institute of the University Lübeck, Germany

^2^ MRC Human Genetics Unit, Institute of Genetics and Molecular Medicine, Edinburgh

^3^ Institute for Clinical Molecular Biology, Christian-Albrechts-University Kiel, Kiel, Germany

^4^ Genetic Epidemiology Unit, Departments of Epidemiology and Clinical Genetics, Erasmus MC, 3000 CA Rotterdam, the Netherlands

^5^ Department of Genetics and Pathology, Rudbeck laboratory, Uppsala University, SE-751 85, Uppsala, Sweden

^6^ Centre for Population Health Sciences, University of Edinburgh Medical School, Teviot Place, Edinburgh EH8 9AG, Scotland, UK

^7^ Andrija Stampar School of Public Health, University of Zagreb Medical School, Rockefellerova 4, 10000 Zagreb, Croatia

^8^ Gen-info Ltd, Ruzmarinka 17, 10000 Zagreb, Croatia

^9^ popgen biobank, Christian-Albrechts-University Kiel, Kiel, Germany

10 Institute for Experimental Medicine, Christian-Albrechts University Kiel, 24105 Kiel,Germany.

^11^ Croatian Centre for Global Health, University of Split Medical School, Soltanska 2, 21000 Split, Croatia

^12^ Institute for Clinical Chemistry and Laboratory Medicine, Regensburg University Medical Center, D-93053 Regensburg, Germany

^13^ Institute of Human Genetics, Technical University of Munich, Munich, Germany

^14^ Institute of Human Genetics, Helmholtz Zentrum München, German Research Center for Environmental Health (GmbH), Ingolstaedter Landstr. 1, D-85764 Neuherberg, Germany

^15^ Hemodialysis Unit, Hospital of Merano, Merano, Italy

^16^ Institute of Medical Informatics and Statistics, Christian-Albrechts-University, Kiel, Germany

^17^ Department of Neurology, University of Lübeck, Lübeck, Germany

^18^ Department of Neurology, Central Hospital, Bolzano, Italy

*Corresponding authors

**Table S1.** Comprehensive association findings for all 29 candidate regions#

| **Genomic region** | **Marker characteristics** | | | |  | **Meta-analysis results** | | **Heterogeneity** | |
| --- | --- | --- | --- | --- | --- | --- | --- | --- | --- |
| **Locus (Chromosome;** bp-bp**)** | **SNP** | **position** | **A1/A2 (Ref. All.)** | **Ref. All. Freq.** | **N pop.** | **Beta (SE)** | **p value** | **I^2^** | **p value** |
| IL28RA  (**1**; 24,353,234-24,495,393) | rs11249039 | 24,425,422 | A/G (G) | 0.34 | 5 | –0.08 (0.02) | 8.3×10^–5^ | 0.00 | 0.90 |
|  | rs10903062 | 24,429,343 | A/G (A) | 0.37 | 5 | –0.09 (0.02) | 2.6×10^–5^ | 0.00 | 0.62 |
|  | rs7517309 | 24,445,393 | A/G (A) | 0.38 | 5 | –0.09 (0.02) | 2.8×10^–5^ | 0.00 | 0.72 |
| CSMD2  (**1**; 33,752,196-34,457,310) | rs1149133 | 34,253,758 | C/T (C) | 0.37 | 1 | 0.15 (0.04) | 4.3×10^–4^ | NA | NA |
|  | rs297804 | 34,443,942 | A/G (A) | 0.27 | 5 | 0.08 (0.02) | 4.9×10^–4^ | 51.75 | 0.08 |
| AGBL4  (**1**; 48,825,047-49,283,906) | rs1484406 | 48,962,821 | A/G (G) | 0.28 | 5 | –0.07 (0.02) | 8.9×10^–4^ | 59.06 | 0.05 |
|  | rs4926749 | 48,975,936 | A/C (C) | 0.30 | 5 | –0.09 (0.02) | 5.8×10^–5^ | 43.51 | 0.14 |
| FMO6P  (**1**; 169,373,503-169,397,326) | rs2272797 | 169,385,464 | A/G (A) | 0.14 | 5 | 0.09 (0.03) | 9.6×10^–4^ | 0.00 | 0.91 |
|  | rs7889839 | 169,387,797 | A/G (G) | 0.14 | 5 | 0.09 (0.03) | 9.7×10^–4^ | 0.00 | 0.91 |
|  | rs7886938 | 169,387,892 | A/G (A) | 0.14 | 5 | 0.10 (0.03) | 7.0×10^–4^ | 0.00 | 0.86 |
| COL4A3  (**2**; 227,737,525-227,887,751) | rs2396463 | 227,753,236 | A/C (A) | 0.16 | 5 | 0.13 (0.03) | 1.5×10^–6^ | 0.00 | 0.93 |
|  | rs2204862 | 227,760,844 | A/G (G) | 0.14 | 5 | 0.11 (0.03) | 4.8×10^–5^ | 0.00 | 0.86 |
| Gene Desert 1  (**3**; 106,037,503-106,171,589) | rs1344861 | 106,087,503 | A/C (C) | 0.31 | 5 | –0.07 (0.02) | 7.9×10^–4^ | 0.00 | 0.90 |
|  | rs6777398 | 106,106,150 | A/G (A) | 0.13 | 5 | 0.10 (0.03) | 2.8×10^–4^ | 0.00 | 0.50 |
|  | rs4895116 | 106,121,589 | A/G (A) | 0.13 | 5 | 0.11 (0.03) | 1.6×10^–4^ | 0.00 | 0.58 |
| GOLGB1-ILDR1  (**3**; 122,864,738-123,223,720) | rs12631803 | 122,915,874 | C/T (T) | 0.11 | 5 | –0.11 (0.03) | 4.9×10^–4^ | 52.03 | 0.08 |
|  | rs11919421 | 122,922,282 | A/C (A) | 0.11 | 5 | –0.11 (0.03) | 3.9×10^–4^ | 51.00 | 0.09 |
|  | rs10511407 | 123,051,285 | C/T (T) | 0.12 | 5 | –0.10 (0.03) | 8.5×10^–4^ | 0.00 | 0.43 |
|  | rs1881998 | 123,096,865 | C/T (C) | 0.13 | 5 | –0.11 (0.03) | 2.1×10^–4^ | 0.00 | 0.54 |
|  | rs4602434 | 123,171,325 | C/T (T) | 0.08 | 5 | –0.13 (0.04) | 9.1×10^–4^ | 0.00 | 0.76 |
|  | rs12489988 | 123,206,390 | A/G (A) | 0.13 | 5 | –0.12 (0.03) | 4.4×10^–5^ | 0.00 | 0.68 |
| Gene Desert 2  (**3**; 181,992,024-182,105,425) | rs13067678 | 182,042,024 | A/G (A) | 0.44 | 5 | –0.07 (0.02) | 2.3×10^–4^ | 0.00 | 0.76 |
|  | rs9838828 | 182,046,241 | G/T (T) | 0.08 | 5 | –0.14 (0.04) | 1.0×10^–4^ | 0.00 | 0.65 |
|  | rs6763026 | 182,053,014 | C/T (T) | 0.16 | 5 | 0.10 (0.03) | 2.5×10^–4^ | 0.14 | 0.43 |
|  | rs1401974 | 182,055,425 | A/G (G) | 0.44 | 5 | –0.07 (0.02) | 2.1×10^–4^ | 0.00 | 0.75 |
| SLC39A8  (**4**; 103,401,844-103,485,371) | rs151401 | 103,409,151 | A/G (G) | 0.19 | 4 | 0.10 (0.03) | 3.4×10^–4^ | 2.82 | 0.36 |
|  | rs10014145 | 103,419,600 | A/G (G) | 0.29 | 5 | 0.08 (0.02) | 1.0×10^–4^ | 0.00 | 0.71 |
| PAPSS1  (**4**; 108,754,271-108,934,028) | rs2522425 | 108,837,681 | C/T (T) | 0.06 | 5 | –0.14 (0.04) | 5.0×10^–4^ | 0.04 | 0.40 |
|  | rs16780 | 108,862,402 | C/T (C) | 0.08 | 5 | –0.12 (0.04) | 7.7×10^–4^ | 0.00 | 0.51 |
|  | rs2522447 | 108,884,028 | G/T (T) | 0.09 | 5 | –0.13 (0.04) | 3.9×10^–4^ | 0.00 | 0.56 |
| DAB2  (**5**; 39,320,758-39,461,092) | rs700233 | 39,400,311 | A/G (A) | 0.40 | 5 | 0.07 (0.02) | 7.6×10^–4^ | 0.00 | 0.48 |
|  | rs835223 | 39,417,114 | A/G (A) | 0.41 | 5 | 0.07 (0.02) | 6.8×10^–4^ | 0.00 | 0.56 |
|  | rs10512696 | 39,459,664 | A/G (A) | 0.39 | 5 | 0.07 (0.02) | 3.6×10^–4^ | 0.00 | 0.87 |
| Gene Desert 3  (**5**; 166,074,316-166,360,859) | rs9313344 | 166,124,316 | A/G (G) | 0.42 | 5 | –0.07 (0.02) | 5.2×10^–4^ | 32.35 | 0.24 |
|  | rs244436 | 166,306,481 | G/T (G) | 0.33 | 5 | 0.09 (0.02) | 4.7 ×10^–5^ | 38.30 | 0.16 |
|  | rs2910281 | 166,310,859 | A/G (A) | 0.21 | 5 | 0.08 (0.02) | 7.6 ×10^–4^ | 0.00 | 0.51 |
| FAM44B  (**5**; 172,967,146-173,068,153) | rs258873 | 172,978,208 | A/C (C) | 0.42 | 5 | –0.08 (0.02) | 3.9 ×10^–5^ | 0.00 | 0.66 |
|  | rs17076219 | 172,998,363 | C/T (T) | 0.10 | 5 | –0.11 (0.03) | 8.1 ×10^–4^ | 0.00 | 0.60 |
|  | rs10516102 | 173,018,153 | C/T (T) | 0.06 | 5 | –0.14 (0.04) | 8.4 ×10^–4^ | 0.00 | 0.94 |
| Gene Desert 4  (**6**; 81,126,957-81,409,053) | rs9352834 | 81,176,957 | C/T (C) | 0.08 | 5 | 0.12 (0.04) | 7.5 ×10^–4^ | 44.41 | 0.13 |
|  | rs515347 | 81,233,946 | A/G (G) | 0.08 | 5 | 0.12 (0.04) | 7.1 ×10^–4^ | 44.91 | 0.13 |
|  | rs1454447 | 81,359,053 | A/G (A) | 0.08 | 5 | 0.14 (0.04) | 1.3 ×10^–4^ | 55.19 | 0.06 |
| GABRR2-UBE2J1  (**6**; 90,023,958-90,178,497) | rs3777514 | 90,077,300 | A/C (A) | 0.20 | 5 | 0.09 (0.02) | 4.5 ×10^–4^ | 2.20 | 0.38 |
|  | rs2064831 | 90,089,661 | C/T (C) | 0.18 | 5 | 0.10 (0.03) | 7.2 ×10^–5^ | 6.73 | 0.29 |
|  | rs1998576 | 90,099,856 | A/G (A) | 0.18 | 5 | 0.10 (0.03) | 8.6 ×10^–5^ | 9.70 | 0.38 |
|  | rs7744005 | 90,170,860 | G/T (T) | 0.22 | 5 | 0.08 (0.02) | 3.6 ×10^–4^ | 0.00 | 0.54 |
| Gene Desert 5  (**6**; 150,831,347-150,960,792) | rs9372065 | 150,881,347 | C/T (C) | 0.33 | 5 | –0.07 (0.02) | 9.1 ×10^–4^ | 0.00 | 0.54 |
|  | rs1492574 | 150,910,136 | C/T (C) | 0.40 | 5 | 0.07 (0.02) | 2.8 ×10^–4^ | 0.00 | 0.84 |
|  | rs9371462 | 150,910,792 | A/G (G) | 0.27 | 5 | 0.08 (0.02) | 5.7 ×10^–4^ | 29.16 | 0.28 |
| CACNA2D1  (**7**; 81,349,193-81,910,967) | rs1229487 | 81,399,193 | G/T (G) | 0.26 | 4 | 0.10 (0.03) | 7.0 ×10^–5^ | 0.00 | 0.85 |
|  | rs2075023 | 81,418,189 | A/C (C) | 0.18 | 2 | –0.14 (0.04) | 3.8 ×10^–4^ | 58.99 | 0.12 |
| YWHAZ  (**8**; 102,000,090-102,117,683) | rs2386922 | 102,044,655 | G/T (G) | 0.41 | 5 | 0.07 (0.02) | 8.9 ×10^–4^ | 0.02 | 0.32 |
|  | rs4002957 | 102,057,402 | A/G (A) | 0.45 | 5 | 0.07 (0.02) | 5.9 ×10^–4^ | 14.13 | 0.32 |
|  | rs3019266 | 102,067,683 | C/T (T) | 0.36 | 5 | 0.07 (0.02) | 6.2 ×10^–4^ | 30.23 | 0.19 |
| COLEC1  (**8**; 119,906,104-120,188,388) | rs2055101 | 119,956,104 | A/G (G) | 0.49 | 5 | 0.07 (0.02) | 8.3 ×10^–4^ | 41.24 | 0.14 |
|  | rs11573829 | 120,028,804 | A/G (A) | 0.42 | 4 | –0.08 (0.02) | 7.7 ×10^–4^ | 21.12 | 0.26 |
|  | rs2326193 | 120,098,890 | A/C (A) | 0.42 | 5 | –0.07 (0.02) | 5.1 ×10^–4^ | 11.80 | 0.34 |
|  | rs6469804 | 120,114,010 | C/T (C) | 0.42 | 5 | –0.07 (0.02) | 5.2 ×10^–4^ | 12.49 | 0.33 |
| ASAP1-DDEF1  (**8**; 131,133,535-131,483,399) | rs4639572 | 131,187,840 | C/T (C) | 0.06 | 5 | 0.16 (0.04) | 4.2 ×10^–5^ | 0.00 | 0.67 |
|  | rs11778881 | 131,360,369 | C/T (C) | 0.05 | 4 | 0.18 (0.05) | 1.8 ×10^–4^ | 0.00 | 0.98 |
| COL22A1  (**8**; 139,669,660-139,995,418) | rs4075073 | 139,808,143 | A/C (A) | 0.37 | 5 | 0.07 (0.02) | 9.8 ×10^–4^ | 0.00 | 0.92 |
|  | rs4588898 | 139,815,390 | A/G (A) | 0.30 | 5 | 0.08 (0.02) | 2.1 ×10^–4^ | 0.00 | 0.92 |
|  | rs9324496 | 139,833,578 | C/T (T) | 0.34 | 5 | 0.07 (0.02) | 4.6 ×10^–4^ | 0.00 | 0.54 |
|  | rs2873682 | 139,840,693 | A/G (A) | 0.30 | 3 | 0.09 (0.03) | 5.2 ×10^–4^ | 0.00 | 0.98 |
| Gene Desert 6  (**9**; 127,850,525-127,970,098) | rs2080558 | 127,900,525 | A/C (A) | 0.10 | 5 | 0.13 (0.03) | 6.6 ×10^–5^ | 0.00 | 0.95 |
|  | rs7871394 | 127,920,098 | A/G (A) | 0.17 | 5 | 0.09 (0.03) | 6.8 ×10^–4^ | 0.00 | 0.92 |
| ODZ2  (**11**; 78,589,476-78,731,499) | rs2007588 | 78,639,476 | G/T (T) | 0.39 | 5 | –0.07 (0.02) | 2.8 ×10^–4^ | 0.00 | 0.91 |
|  | rs487345 | 78,681,499 | C/T (C) | 0.22 | 5 | –0.11 (0.02) | 9.6 ×10^–6^ | 0.00 | 0.95 |
| HNT  (**11**; 130,745,581-131,711,926) | rs4083232 | 131,179,197 | A/G (G) | 0.27 | 5 | –0.08 (0.02) | 2.7 ×10^–4^ | 0.01 | 0.43 |
|  | rs3740822 | 131,191,424 | A/G (A) | 0.48 | 5 | –0.07 (0.02) | 5.1 ×10^–4^ | 0.03 | 0.42 |
|  | rs483097 | 131,195,776 | C/T (T) | 0.22 | 5 | –0.09 (0.02) | 5.8 ×10^–5^ | 0.00 | 0.78 |
| SYT1  (**12**; 77,955,428-78,079,267) | rs10506807 | 78,005,428 | C/T (C) | 0.13 | 5 | –0.11 (0.03) | 7.0 ×10^–5^ | 21.88 | 0.17 |
|  | rs12300068 | 78,005,502 | A/G (A) | 0.13 | 5 | –0.12 (0.03) | 5.7 ×10^–5^ | 24.11 | 0.17 |
|  | rs11112829 | 78,029,267 | C/T (C) | 0.13 | 5 | –0.11 (0.03) | 7.6 ×10^–5^ | 19.94 | 0.18 |
| KCNK10  (**14**; 87,720,993-87,863,004) | rs1940545 | 87,762,395 | C/T (T) | 0.24 | 5 | –0.08 (0.02) | 3.9 ×10^–4^ | 0.00 | 0.53 |
|  | rs7140315 | 87,765,095 | A/G (G) | 0.24 | 5 | –0.08 (0.02) | 5.4 ×10^–4^ | 0.00 | 0.59 |
|  | rs10483992 | 87,785,125 | C/T (C) | 0.23 | 5 | –0.09 (0.02) | 2.1 ×10^–4^ | 41.60 | 0.16 |
|  | rs10483995 | 87,786,360 | A/C (C) | 0.21 | 5 | –0.09 (0.02) | 3.4 ×10^–4^ | 57.01 | 0.05 |
| HSP90AA-CINP  (**14**; 101,616,828-101,899,006) | rs7157967 | 101,655,966 | C/T (C) | 0.12 | 5 | –0.12 (0.03) | 7.9 ×10^–5^ | 0.00 | 0.56 |
|  | rs11160674 | 101,757,609 | C/T (C) | 0.13 | 5 | –0.13 (0.03) | 1.6 ×10^–5^ | 48.26 | 0.11 |
|  | rs7157909 | 101,803,964 | C/T (C) | 0.22 | 5 | –0.08 (0.02) | 4.0 ×10^–4^ | 0.00 | 0.88 |
|  | rs4601978 | 101,820,830 | C/T (T) | 0.22 | 5 | –0.08 (0.02) | 5.1 ×10^–4^ | 0.00 | 0.90 |
|  | rs3759558 | 101,844,013 | A/G (A) | 0.16 | 5 | –0.10 (0.03) | 1.2 ×10^–4^ | 0.00 | 0.61 |
|  | rs7158731 | 101,862,139 | C/T (C) | 0.17 | 5 | –0.09 (0.03) | 3.1 ×10^–4^ | 0.00 | 0.56 |
| Gene Desert 7  (**16**; 13,526,010-13,738,436) | rs4780525 | 13,576,010 | C/T (C) | 0.17 | 4 | 0.10 (0.03) | 7.7 ×10^–4^ | 0.00 | 0.45 |
|  | rs3916993 | 13,675,213 | A/G (G) | 0.04 | 5 | 0.07 (0.02) | 4.3 ×10^–4^ | 0.00 | 0.54 |
|  | rs2051545 | 13,680,100 | G/T (T) | 0.37 | 5 | 0.07 (0.02) | 9.8 ×10^–4^ | 0.00 | 0.48 |
|  | rs2097289 | 13,688,436 | A/G (G) | 0.38 | 5 | 0.07 (0.02) | 8.7 ×10^–4^ | 0.00 | 0.48 |
| DMD  (**X**; 31,047,266-33,267,647) | rs12390820 | 32,887,478 | A/G (G) | 0.21 | 5 | 0.07 (0.02) | 5.9 ×10^–4^ | 0.00 | 0.97 |
|  | rs7052798 | 32,888,063 | A/C (A) | 0.21 | 5 | 0.07 (0.02) | 5.9 ×10^–4^ | 0.00 | 0.97 |
|  | rs5972721 | 32,891,871 | A/G (A) | 0.21 | 5 | 0.07 (0.02) | 8.2 ×10^–4^ | 0.00 | 0.92 |

^#^ association models were adjusted for age and sex.

**List of abbreviations used**: **Ref. All.**: Reference Allele; **Ref. All. Freq.**: Reference Allele Frequency; **N pop.**: number of populations with the specific SNP genotyped; **Beta, SE**: beta and standard errors, in standard deviations; **I^2^**: Heterogeneity statistics; **Heterogeneity p value**: p value for the homogeneity test; p<0.10 means the hypothesis of homogeneity is rejected.

**Table S2.** Results of the replication analysis. Regions are ordered by increasing replication p value.

| **Genomic region** | **popgen** | | **Korcula** | | **Replication meta-analysis** | **FDR analysis** |
| --- | --- | --- | --- | --- | --- | --- |
| **Locus** | **n_1_/N_1_** | **min p_1_** | **n_2_/N_2_** | **min p_2_** | **Fisher**  **p value** | **Significance threshold** |
| COL22A1 | 19/140 | 0.001 | 11/71 | 0.006 | 1.0×10^–6^ | 0.0017 |
| SYT1 | 9/31 | 1.5x10^–5^ | 0/9 | 0.112 | 1.7×10^–4^ | 0.0034 |
| GABRR2-UBE2J1 | 8/37 | 0.002 | 0/27 | 0.062 | 3.6×10^–3^ | 0.0052 |
| CACNA2D1 | 8/161 | 0.007 | 10/78 | 0.005 | 0.021 | 0.0069 |
| FAM44B | 2/34 | 0.027 | 4/16 | 0.001 | 0.024 | 0.0086 |
| DMD | 25/639 | 1.0×10^–4^ | 29/401 | 2.7×10^–4^ | 0.131 | 0.0103 |
| YWHAZ | 3/16 | 0.019 | 0/11 | 0.080 | 0.178 | 0.0121 |
| COL4A3 | 9/102 | 0.002 | 1/31 | 0.027 | 0.216 | 0.0137 |
| Gene Desert 1 | 1/26 | 0.008 | 3/20 | 0.014 | 0.216 | 0.0155 |
| DAB2 | 2/31 | 0.025 | 2/16 | 0.040 | 0.301 | 0.0172 |
| SLC39A8 | 1/25 | 0.044 | 2/13 | 0.016 | 0.325 | 0.0190 |
| COLEC1 | 7/89 | 0.022 | 2/49 | 0.038 | 0.357 | 0.0207 |
| HSP90AA-CINP | 2/30 | 0.004 | 2/20 | 0.024 | 0.370 | 0.0224 |
| CSMD2 | 14/268 | 0.006 | 8/122 | 0.012 | 0.387 | 0.0241 |
| ODZ2 | 4/72 | 0.031 | 2/37 | 0.046 | 0.627 | 0.0259 |
| Gene Desert 3 | 5/77 | 0.011 | 1/42 | 0.005 | 0.663 | 0.0276 |
| ASAP1-DDEF1 | 4/87 | 0.019 | 1/30 | 0.048 | 0.847 | 0.0293 |
| Gene Desert 7 | 0/106 | 0.086 | 2/34 | 0.010 | 0.855 | 0.0310 |
| Gene Desert 4 | 0/70 | 0.080 | 1/24 | 0.036 | 0.952 | 0.0328 |
| Gene Desert 5 | 2/50 | 0.045 | 0/24 | 0.084 | 0.957 | 0.0345 |
| Gene Desert 6 | 0/45 | 0.081 | 1/27 | 0.047 | 0.966 | 0.0362 |
| GOLGB1-ILDR1 | 0/52 | 0.091 | 1/35 | 0.022 | 0.985 | 0.0379 |
| KCNK10 | 1/48 | 0.038 | 0/21 | 0.088 | 0.996 | 0.0399 |
| HNT | 8/405 | 0.017 | 5/169 | 0.011 | 0.997 | 0.0412 |
| IL28RA | 1/65 | 0.018 | 0/22 | 0.084 | 0.999 | 0.0431 |
| AGBL4 | 4/227 | 0.007 | 0/43 | 0.079 | 1.000 | 0.0448 |
| FMO6P | 0/13 | 0.517 | 0/6 | 0.150 | 1.000 | 0.0465 |
| Gene Desert 2 | 0/7 | 0.309 | 0/11 | 0.096 | 1.000 | 0.0483 |
| PAPSS1 | 0/31 | 0.063 | 0/17 | 0.146 | 1.000 | 0.0500 |

**List of abbreviations used**: **Bp**: Base-pairs; **n_1_/N_1_**, **n_2_/N_2_**: no. of tests with p value ≤ 0.05 to the no. of tests performed in popgen (1) and Korcula (2), respectively; **min p_1_**, **min p_2_**: minimum p value observed in popgen and Korcula, respectively; **Fisher p**: p value from the Fisher's combined probability test; **FDR**: false discovery rate.

**Table S3.** Replication of loci associated with estimated glomerular filtration rate (GFR) in Köttgen *et al.* [18].*

| **Locus name** | **SNP** | **p value (CHARGE)** | **p value (Eurospan)** | **Region-specific p value** |
| --- | --- | --- | --- | --- |
| UMOD | rs4293393 | 4.9×10^–11^ | 3.9×10^–4^ | 1.3×10^–4^ |
| SPATA | rs2461700** | 3.9×10^–7^ | 5.0×10^–4^ | 1.2×10^–3^ |
| SCHROOM3 | rs4859682 | 3.0×10^–7^ | 1.7×10^–3^ | 0.50 |
| JAG1 | rs6040055 | 1.0×10^–8^ | 0.52 | 1.00 |

*Threshold for statistical significance, under Bonferroni correction for multiple testing: *0.0125*.

**This SNP was not available in the Eurospan consortium analysis. The proxy SNP rs1153860 was used instead (the two SNPs in the HapMap-CEU database, Phase III/Release 2, were in perfect linkage disequilibrium, that, is *r^2^* = 1.00).
